# Impact of Advanced Platelet-Rich Fibrin on Early Bone Healing After Endodontic Microsurgery: A Randomized Controlled Trial

**DOI:** 10.3390/diagnostics15050516

**Published:** 2025-02-20

**Authors:** Aleksandra Karkle, Laura Neimane, Maksims Zolovs, Matiss Dambergs, Dita Meistere, Anete Vaskevica, Anda Slaidina

**Affiliations:** 1 Department of Conservative Dentistry and Oral Health, Riga Stradins University, LV-1007 Riga, Latvia; 2 RSU Institute of Stomatology, LV-1007 Riga, Latvia; 3 Statistics Unit, Riga Stradins University, LV-1007 Riga, Latvia; 4 Institute of Life Sciences and Technology, Daugavpils University, LV-5401 Daugavpils, Latvia; 5 Department of Orthodontics, Riga Stradins University, LV-1007 Riga, Latvia; 6 Department of Prosthetic Dentistry, Riga Stradins University, LV-1007 Riga, Latvia

**Keywords:** apical surgery, advanced platelet-rich fibrin, bone regeneration, CBCT

## Abstract

**Background:** Apical surgery can address periapical pathologies when conventional endodontic treatments fail. Advanced platelet-rich fibrin (A-PRF) has emerged as a regenerative material with the potential to enhance healing in periapical surgery. In this study, we evaluated the effect of A-PRF on periapical lesion healing using cone beam computed tomography (CBCT). **Methods:** A randomized controlled trial at Rīga Stradiņš University Institute of Stomatology included 43 participants (15 males, 28 females; mean age: 44 years) with periradicular lesions. Participants were divided into experimental and control groups. Baseline and follow-up CBCT imaging assessed lesion volumes at 6 and 12 months post-surgery. **Results:** Mean lesion volumes significantly decreased from 431.4 mm^3^ at baseline to 102.8 mm^3^ at 6 months and 49.2 mm^3^ at 12 months (*p* < 0.001). A-PRF did not significantly reduce the lesion sizes compared to the controls (*p* = 0.043), but was associated with reduced inflammation and enhanced soft tissue healing. Key confounders included gender and baseline lesion volume, with males exhibiting smaller lesion sizes postoperatively. **Conclusions:** The results suggest that A-PRF may not offer a significant advantage in terms of reducing defect size or improving radiographic resolution.

## 1. Introduction

Apical surgery is used to address periapical dental pathologies when conventional endodontic methods fail to achieve the desired results or cannot be applied for various reasons. It is regarded as a standard procedure in oral surgery and often serves as the last opportunity to preserve a tooth affected by periapical issues, particularly when traditional endodontic treatments or retreatments have been proven to be ineffective or unfeasible [1]. The primary goal of an apicoectomy is to prevent bacterial leakage from the root canal into the surrounding periradicular tissue by filling the resected root canal with a dense and impermeable material [2]. The cause of a periapical infection can be intraradicular or extraradicular [3]. Regenerative medicine has been praised as a “biological solution to biological and medical problems”, and growth factors are essential to its effectiveness [4]. Different regenerative techniques have been promoted to inhibit the apical movement of junctional epithelium in these situations, including the use of guided tissue regeneration (GTR) barrier membranes and grafts, alongside endodontic microsurgery [5]. Autologous platelet concentrates have recently come to light as cutting-edge regenerative materials with great promise for accelerating tissue regeneration and wound healing through the high-concentration release of growth factors [5]. The advantages of platelet concentrates in periapical surgery have been the subject of numerous studies [6,7,8].

Research in this field focuses on discovering methods to regenerate lost bone mass, while also placing significant emphasis on the role of vitamin D and cholesterol. Vitamin D, as a steroid hormone, plays a vital role in bone mineralization, which is crucial for healing and regeneration [9]. Given its impact on bone metabolism and immune regulation, vitamin D is particularly relevant in oral surgery, with studies highlighting its potential to enhance postoperative healing, improve osseointegration in dental implants, and support bone regeneration [10]. Elevated cholesterol and triglyceride levels can negatively impact the immune system and bone health, leading to reduced bone mineral density [11,12]. Hyperlipidemia has been shown to increase osteoclast numbers, inhibit osteoblastic activity, and disrupt bone remodeling, resulting in decreased bone mass [13]. Due to these effects, hyperlipidemia may also hinder bone grafting and oral surgery, as the host’s bone quality and healing capacity are essential for successful regeneration [14,15,16,17].

Research on advanced imaging techniques has demonstrated that methods such as synchrotron radiation micro-computed tomography (SRμCT) and cone beam micro/nano X-ray tomography provide superior precision when characterizing the three-dimensional structure of bone. These techniques offer higher contrast and greater sensitivity to subtle variations in tissue density due to the enhanced uniformity of the X-ray beam [18]. Nevertheless, clinical studies assessing healing results have primarily relied on two-dimensional imaging methods, such as periapical radiography [19,20,21,22]. While these methods provide valuable information, cone beam computed tomography (CBCT) offers significant advantages in three-dimensional imaging, enhancing diagnostic accuracy and improving clinical decision-making. In clinical practice, CBCT remains the gold standard for X-ray imaging [23]. It is widely used in clinical trials to assess bone regeneration, guide surgical planning, and determine the ideal angulation with high precision [24]. CBCT improves sensitivity and accuracy when identifying bone abnormalities, particularly in sagittal and axial sections, making it an essential tool for evaluating the cortical bone structure and healing outcomes [25].

The objective of this randomized controlled trial was to evaluate the impact of tissue regeneration with platelet-rich fibrin on the healing of periapical lesions, utilizing periapical radiographs (PA) and cone beam computed tomography (CBCT) imaging.

The hypotheses are as follows:

**H0.**  *The application of advanced platelet-rich fibrin (A-PRF) does not significantly enhance early bone regeneration compared to apical surgery performed without the use of A-PRF*.

**H1.**  *The application of advanced platelet-rich fibrin (A-PRF) enhances early bone regeneration compared to apical surgery performed without the use of A-PRF*.

## 2. Materials and Methods

**Figure 1:**
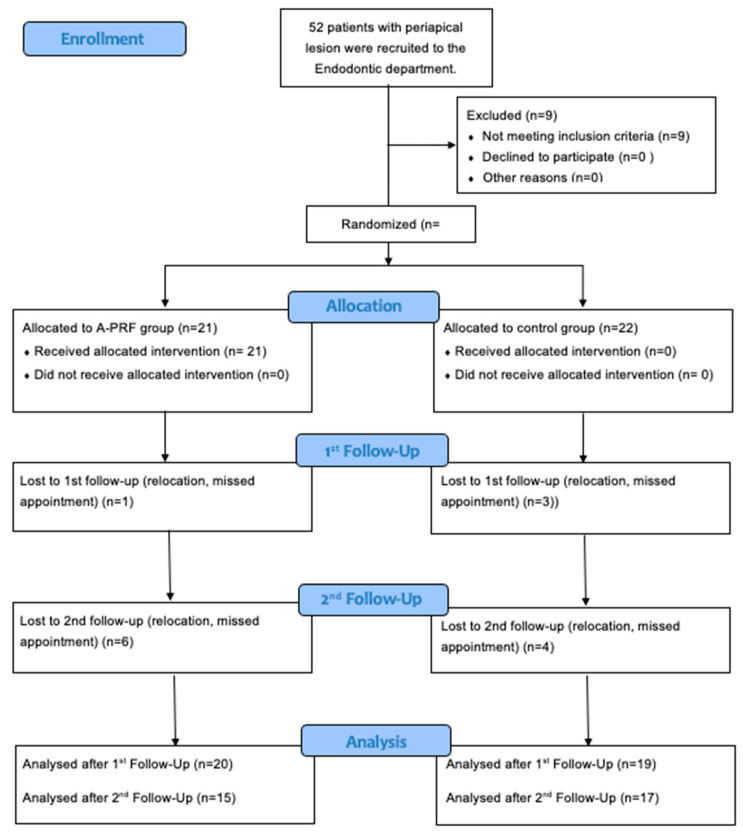
Consort flow-chart 2010.

This study was a randomized clinical trial. The study was registered online on “www.clinicaltrial.gov” with the registration number NCT06734962. As illustrated in the consort 2010 flow-chart (Figure 1), 43 patients (28 females and 15 males) were enrolled in the study at the Rīga Stradiņš University Institute of Stomatology Endodontic clinics and in the RSU Department of Conservative Dentistry and Oral Health, Riga and Oral, and Maxillofacial Surgery Clinic, Latvia. This study was conducted in accordance with the Declaration of Helsinki and was approved by the Ethics Committee of Rīga Stradiņš University (protocol Nr. 22-2/427/2021; date of approval, 11 August 2021). Before participation, patients were offered extensive information about the study’s objectives, design, procedures, and protocols, emphasizing the dedication to ethical research practices. Once they had given their informed consent, the individuals were enrolled in the study.

### 2.1. Inclusion/Exclusion Criteria

The study included patients of both genders, aged from 18 to 78 (mean age: 43.95 ± 14.06 y), who were referred to the Department of Endodontics, Rīga Stradiņš University Institute of Stomatology (Riga, Latvia) for apical surgery. The period from September 2021 to October 2024 was selected. The sample comprised 52 teeth with periapical lesions associated with maxillary and mandibular incisors, canines, and premolars. Criteria for the eligibility of inclusion were adult patients with precisely defined periapical lesions related to maxillary or mandibular incisors, canines, and premolar teeth as a sequel to persistent endodontic infection after root canal treatment/retreatment with/without the sinus tract; root perforations; cases deemed unsuitable for non-surgical endodontic intervention; or trauma clearly indicated for endodontic apical surgery. We excluded patients with lesions unrelated to the root apical area; in possession of vital teeth with radiolucency in the apical region; that were pregnant; younger than 18 years old; with non-restorable teeth; with advanced periodontal disease or uncontrolled systemic health conditions; receiving bisphosphonate therapy; and receiving orthodontic treatment. All patients confirmed that they did not smoke before enrolling in the study. This criterion was implemented to eliminate any potential impact of smoking on the study outcomes, such as impaired healing or altered immune responses, ensuring a more controlled and accurate assessment of the treatment or intervention.

### 2.2. Sample Size Calculation and Patient Selection

A power analysis was conducted to determine the required sample size for a within–between research design (two groups with three repeated measures). Given an alpha level of 0.05, a desired power of 0.95, and a moderate effect size of 0.25, the calculated optimal sample size was 44 patients. The sample size was calculated using G*Power 3.1.9.

### 2.3. Patient Randomization

The block randomization method was used in this clinical trial to ensure that treatment groups were balanced in terms of the number of participants allocated to each group (Figure 1). Participants were enrolled sequentially as they arrived for their scheduled surgeries. A fixed block size of four was used, with the allocation sequence of AABB carefully randomized across blocks to ensure unpredictability. Within each block, two patients were assigned to treatment group A and two to treatment group B, ensuring balanced group sizes. The allocation process was fully blinded to both patients and investigators to avoid selection bias. As patients were enrolled, they were assigned to the next available slot in the randomized sequence, maintaining the integrity of the allocation plan.

### 2.4. Examination

All patients enrolled in this study were scheduled for apical surgery procedures. Each patient underwent clinical and radiological evaluations at baseline, as well as follow-ups at 6 and 12 months using PA X-rays and CBCT imaging. Radiological examinations were performed in a blinded manner by an independent specialist from the Radiology Department, while clinical evaluations were conducted separately in the Endodontic Department by the same operator who performed the apical surgery. A customized form was designed to record each patient’s medical history and clinical examination details, including endodontic tests, pulp vitality assessments, percussion, palpation, pocket measurements, and mobility tests. Before the surgical procedure, each patient was referred to a laboratory for blood tests to assess vitamin D (D3+D2) and cholesterol levels. Blood samples were collected and analyzed in the laboratory using electrochemical luminescence for vitamin D measurement and an enzymatic color reaction for cholesterol.

Vitamin D (D3+D2) Testing: The test was performed on serum or plasma with the following sample tube markings: red, yellow-green, or violet. Blood samples were stored at room temperature (15–25 °C) for up to 8 h, in the refrigerator (2–8 °C) for up to 4 days, and frozen at −20 °C for up to 24 weeks. The reference values for vitamin D (D3+D2) were as follows: optimal, 45.0–55.0 ng/mL; sufficient, >30 ng/mL; insufficient, 20.0–29.9 ng/ mL; critically low, mL; deficient, <19.9 ng/<10 ng/mL. These values align with the “Osteoporosis Clinical Guidelines”.

Total Cholesterol Testing: For the cholesterol analysis, serum was collected in red or yellow tubes without anticoagulants. Samples were stored at room temperature (+15 to +25 °C) or in the refrigerator (+2 to +8 °C) for up to 7 days. The reference value for Total Cholesterol was <5.0 mmol/L. This study provides reference intervals for both vitamin D (D3+D2) and cholesterol levels, contributing valuable information for assessing patient health in clinical settings. Follow-up data were securely stored in a blinded database, with treatment codes inaccessible to both operators and participants. Data entry was conducted by personnel who were unaware of group allocations.

### 2.5. Evaluation Parameter

To ensure accurate and clear images before the PA X-ray procedure, all patients were positioned properly and provided a protective shield to guard against radiation exposure. PA radiographs were taken at baseline and at 6- and 12-month follow-up visits by utilizing a Durr Imaging Plate and Film Holder System (Durr Dental, Bietigheim-Bissingen, Germany) with intraoral phosphor sensor plates (Durr Dental, Bietigheim-Bissingen, Germany) and a Planmeca Prox dental X-ray unit (Planmeca, Helsinki, Finland). VistaSoft software 3.0.31 was used for image interpretation. If necessary, different image processing facilities provided by the VistasScan software system were used to ensure accuracy.

CBCT scans were acquired using the same protocol on a single unit (Veraview 800X, Morita, Osaka, Japan), operating at 102 kV, 5.1 mA. The field of view (FOV) was set at 40 × 40 × 40 mm, with a voxel size of 0.125 mm. To ensure optimal viewing conditions, i-Dixel software Version 2,15, 0 (Morita, Japan) was used for image analysis.

The Dell 5820 Precision workstation, in conjunction with a 23.6-inch LCD Dell monitor boasting a resolution of 1920 × 1080, was used for data evaluation. As a primary outcome, a reduction in lesion volume in mm^3^ was evaluated via a volumetric analysis of CBCT images using the software 26.0 Materialise Mimics (Leuven, Belgium).

As secondary outcomes, the healing of periapical lesions was evaluated by Molven’s criteria (to evaluate the 2D periapical healing in PA images) and by the modified Penn 3D criteria (to evaluate 3D periapical healing in CBCT images).

### 2.6. Surgical Protocol

The surgical procedure included surgical interventions under an operating microscope and magnification loups. Articaine with epinephrine 1:100,000 was used as a local anesthetic. Surgical access included a full-thickness mucoperiosteal flap with vertical incisions. The flap design was selected for apical microsurgery due to its ability to preserve the gingival structure while providing adequate surgical access. Its design prevents gingival recession and papilla retraction, ensuring optimal esthetic outcomes, particularly in the anterior regions. Additionally, the technique minimizes scarring and maintains vascular integrity, which supports faster healing. This flap offers significant advantages in maintaining soft tissue stability and facilitating successful postoperative recovery [26]. A round bur on a low-speed handpiece with constant water irrigation was used to remove the bone around the root apex. All granulomatous tissue was removed by curettage. The root apex was sectioned 3 mm from the anatomical apex. The root apex cavity was performed using ultrasonic tips, and the apical orifice of the resected root was colored with methylene blue. The surgical mirror was used to evaluate the resected root surface and root canal anatomy. A retrograde filling FKG Putty fast set was performed. The bone defect was cleaned with saline. An A-PRF membrane was applied to the PRF test group, but the bone defect for patients in the control group was not filled, and the flap was closed and sutured with 6 × 0 monofilament polypropylene. Monofilament sutures were chosen to minimize tissue trauma and reduce friction during the procedure. They were less likely to harbor bacteria, offered stability and strength, and minimized irritation in surgical areas. After the sutures were applied, the patient was promptly administered a cold compress. The painkiller was prescribed as needed by the patient’s choice. Antibiotics were typically not prescribed unless indicated by the patient’s medical history [27]. Sutures were removed within 48–72 h from surgical intervention [28].

### 2.7. A-PRF Application Protocol

**Figure 2:**
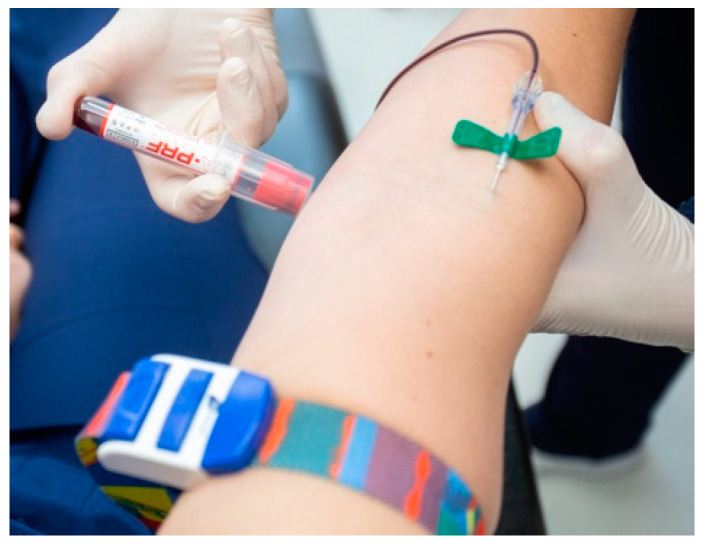
Blood collection from patient’s vein.

**Figure 3:**
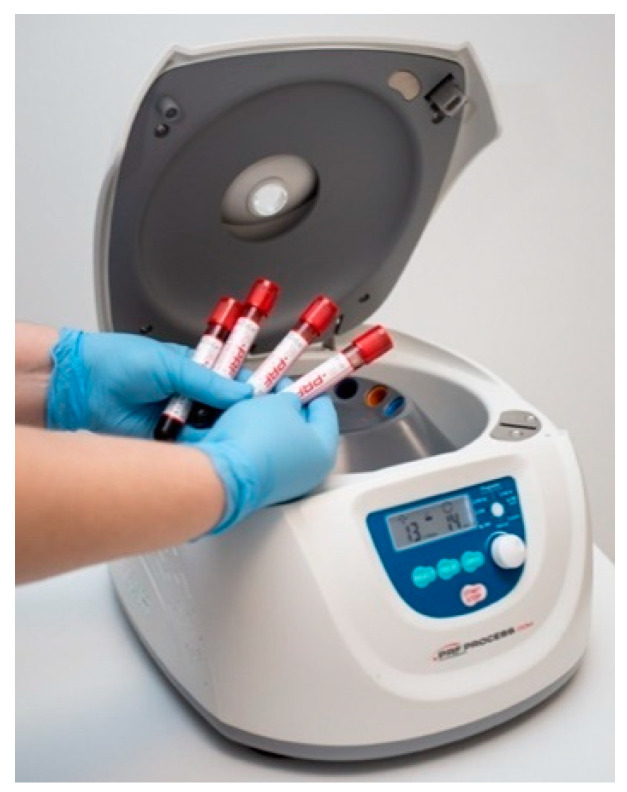
10-milliliter sterile glass vacutainers for venous blood collection.

For the A-PRF preparation and application, Choukroun’s protocol was used [29]. Blood was taken by the anesthesiologist from the patient’s basal vein or vein cephalica (Figure 2). The 10-milliliter sterile glass A-PRF vacutainers devoid of additives (Figure 3) were utilized for venous blood collection from the patient. The blood was promptly centrifuged post-collection, as fibrin polymerization commences upon entry into the tube.

**Figure 4:**
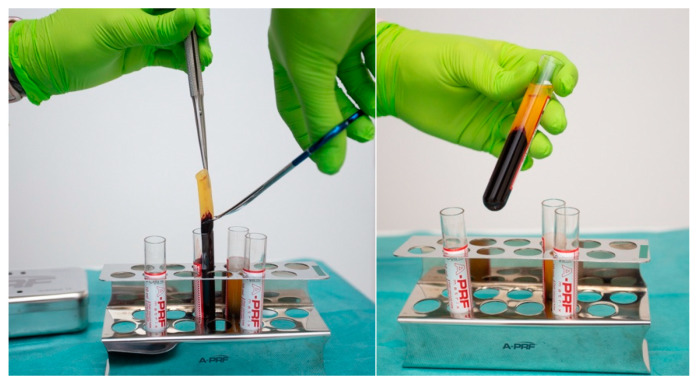
Removing the layer of red blood cells.

**Figure 5:**
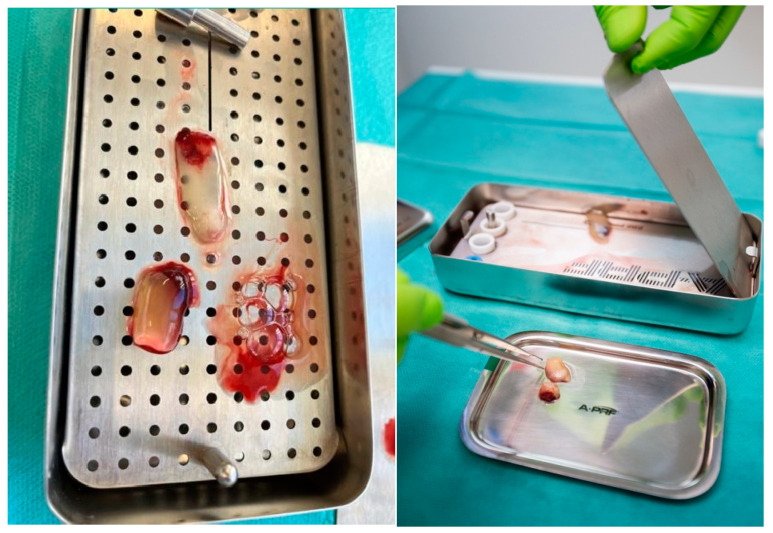
Prepared PRF membrane in PRF box.

Samples were centrifuged in a PRF DUO QUATTRO centrifuge (Nice, France) for 14 min at 1300 revolutions per minute, as per the manufacturer’s recommendation. The blood stratifies into three distinct layers, as follows: platelet-poor plasma (PPP) at the apex, red blood cells (RBCs) at the base, and a fibrin clot of platelet-rich fibrin (PRF) in the intermediate layer. The fibrin clot of PRF was separated using tweezers (Figure 4). The layer of red blood cells was excised, and a membrane press box was used to make an A-PRF membrane by expelling the fluid (Figure 5). An A-PRF clot was applied to the defect area, covering the entire region, including the borders, to ensure complete coverage. The flap was closed, and sutures were imposed.

### 2.8. 2D Radiographic Healing Evaluation

Molven’s criteria were used to evaluate the 2D periapical healing [30] in the following ways (Table 1):

### 2.9. 3D Radiographic Healing Evaluation

**Figure 6:**
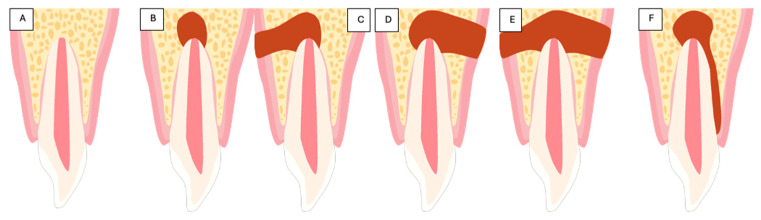
Classification of periapical lesions. (**A**) No periapical lesion; (**B**) the lesion is limited to the periapical area; (**C**) the lesion has eroded the buccal cortex; (**D**) the lesion has eroded the lingual/palatal cortex; (**E**) the lesion has eroded the lingual/palatal and buccal cortex, resulting in a through-and-through (tunnel) defect; (**F**) an apico-marginal lesion is present, with complete denudation of the buccal root surface.

CBCT imaging was used for periapical defect-type evaluation before surgery, utilazing a periapical lesion classification (Figure 6). 

The following modified Penn 3D criteria [21,22,31] were applied to evaluate 3D healing using CBCT imaging (Table 2):

The 2D and 3D radiographic evaluations at baseline and after 6 and 12 months were conducted by a periodontologist (AV) and a general dentist (DM), each with 10 years of CBCT experience. Both observers, trained by a maxillofacial radiologist with 15 years of expertise, reviewed all images twice within a 2-week interval to ensure measurement reliability and to assess interobserver agreement.

### 2.10. Radiography Acquisition: Volumetric Assessment

CBCT image volumetric analyses were performed using Materialise Mimics (Leuven, Belgium). The defect was segmented in axial, sagittal, and coronal views by marking multiple slices in the 2D view, where the change in a defect region appeared the most. As marked pixels not only formed contours, but also an area, interpolation was performed between slices, giving a three-dimensional volume which was measured and used for comparison. After automatic operation, it was corrected in other 2D views to manually control and improve the border of the defect. In the end, the software displays the precise volume in mm^3^ of the marked region.

**Figure 7:**
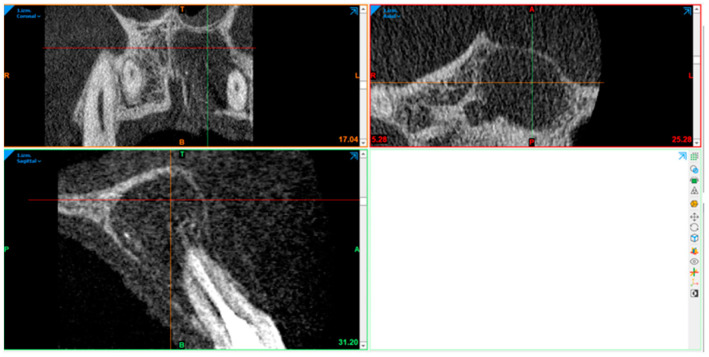
Pre-op: volume measurement with Materialise Mimics.

**Figure 8:**
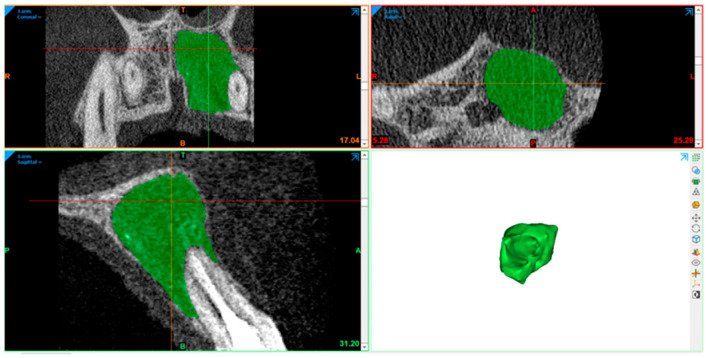
Pre-op: volume measurement with Materialise Mimics. Lesion volume size marked in green.

**Figure 9:**
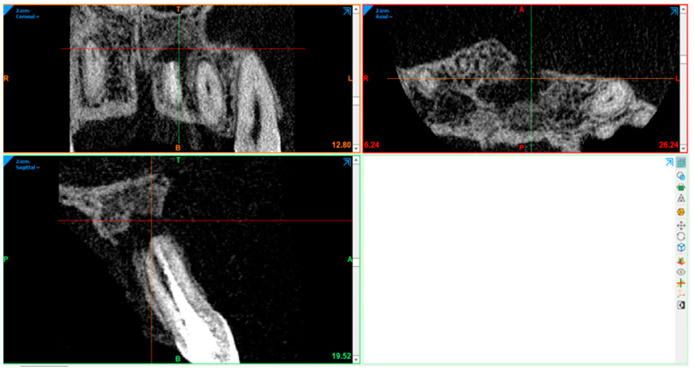
First follow-up volume measurement with Materialise Mimics.

**Figure 10:**
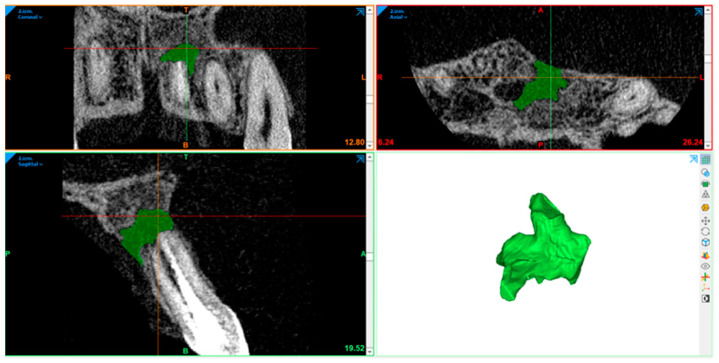
First follow-up volume measurement with Materialise Mimics. Lesion volume size marked in green.

**Figure 11:**
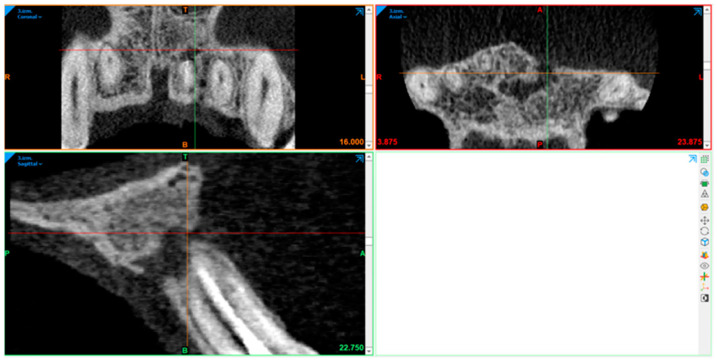
Second follow-up volume measurement with Materialise Mimics.

**Figure 12:**
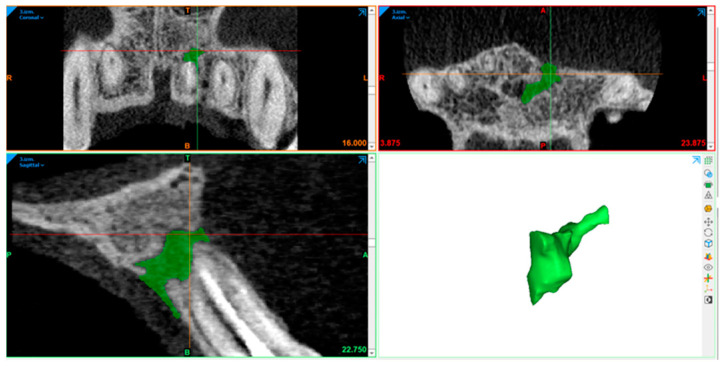
Second follow-up volume measurement with Materialise Mimics. Lesion volume size marked in green.

The defect size was measured preoperatively, at the 6- and 12-month follow-ups (Figure 7, Figure 8, Figure 9, Figure 10, Figure 11 and Figure 12). A single calibrated investigator (MD) performed all volumetric measurements.

### 2.11. Statistical Analysis

To account for the repeated measures of lesion volume over time (baseline, 6 months, and 12 months), a Linear Mixed Model (LMM) was employed to compare the lesion volume between the control and experimental groups. The analysis controlled for potential confounding factors, such as age, gender, baseline lesion volume, vitamin D (D3+D2), Total Cholesterol, and classification of periradicular lesions. To standardize the range of independent variables, Z-score scaling was applied. To ensure the validity and reliability of the models’ results, the assumptions of normality, homoscedasticity, and multicollinearity were tested.

Linear regression was conducted to compare the reduction in lesion volume from baseline to 6 months and from 6 months to 12 months between the control and experimental groups after controlling for age, gender, vitamin D (D3+D2), Total Cholesterol, and classification of periradicular lesions.

Chi-squared tests of homogeneity were employed to compare lesion assessments using Molven’s and the Penn criteria, as well as to compare assessments between the experimental and control groups.

Inter-rater reliability was assessed with Cohen’s Kappa coefficient separately for Molven’s and the Penn criteria. Intrarater volumetric assessment was calculated by using the intraclass correlation coefficient (ICC).

The statistical analysis was performed using the Jamovi software (v 2.5). The result was considered statistically significant if the *p*-value was less than 0.05.

## 3. Results

Molven’s criteria demonstrated substantial inter-rater reliability (Cohen’s kappa = 0.841, *p* < 0.001), with 93.9% agreement between raters. Penn’s criteria showed almost perfect agreement (Cohen’s kappa = 0.915, *p* < 0.001), with 95% agreement between raters. The volumetric assessment showed perfect agreement for ICC = 1.0 (95% CI 0.99–1.0).

This study included 43 participants (15 males, 28 females, mean age 44 years), with participants assigned to experimental (48.8%) and control (51.2%) groups. The majority had maxillary incisors and Type 3 periradicular lesions. The baseline serum vitamin D levels (D3+D2) averaged 36.3 ng/mL, and the Total Cholesterol was 5.15 mmol/L. Notably, there was a significant reduction in lesion volumes, from a mean of 431.4 mm^3^ at baseline to 102.8 mm^3^ at 6 months and 49.2 mm^3^ at 12 months, indicating substantial clinical improvement over time (Table 3).

The Linear Mixed Model was statistically significant for χ^2^(11) = 104.7, *p* < 0.001. After adjusting for potential confounders, such as age, gender, baseline lesion volume, vitamin D (D3+D2), Total Cholesterol, and classification of periradicular lesions, the lesion volume was found to differ significantly between the control and experimental groups (*p* = 0.043). Among the confounders, gender, baseline lesion volume, and classification of periradicular lesions were significantly associated with lesion volume (*p* < 0.05). Specifically, males had a 61.2 mm^3^ lower lesion volume compared to females (95% CI: 21.1–101.4). Additionally, a 1 mm^3^ increase in the baseline lesion volume was associated with a 105.7 mm^3^ increase in the post-surgery lesion volume (95% CI: 84.4–126.9). Detailed results of the Linear Mixed Model are summarized in Table 4. Mean and marginal mean lesion volumes are summarized in Table 5.

**Figure 13:**
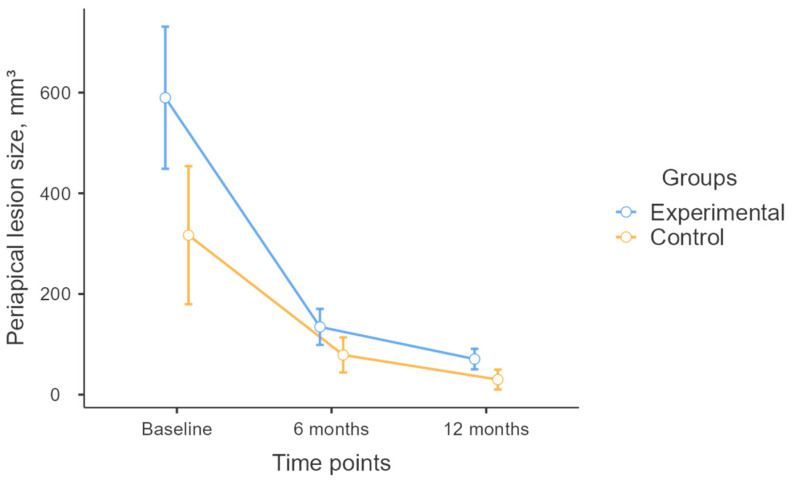
Reduction in periapical lesion size by gender.

**Figure 14:**
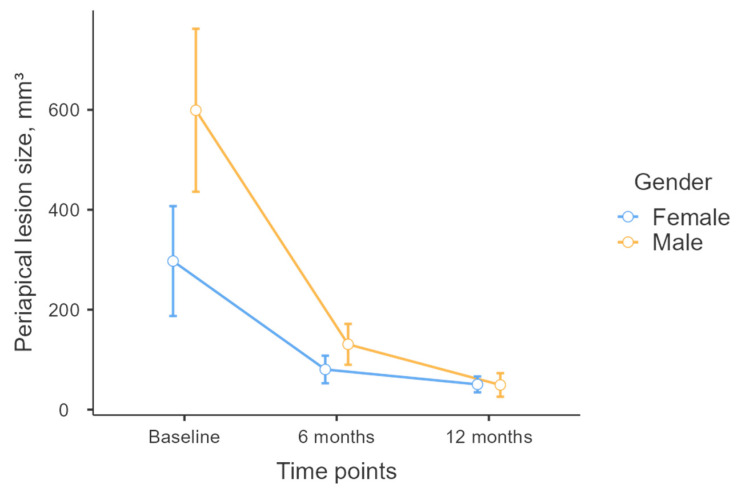
Time-dependent reduction in periapical lesion size: experimental vs. control groups.

Linear regression analysis revealed no significant difference in lesion volume reduction between the control and experimental groups from baseline to 6 months and from 6 months to 12 months (Figure 13) (*p* > 0.05); however, the model identified a significant association between lesion volume reduction and gender (*p* < 0.05). On average, males experienced a 462 mm^3^ greater reduction in lesion volume from baseline to 6 months compared to females. Additionally, males exhibited a 51 mm^3^ greater reduction in lesion volume from 6 months to 12 months compared to females (Figure 14).

A comparison of the lesion assessment using Molven’s and the Penn criteria (Table 6) demonstrated a statistically significant difference between the two methods (*p* < 0.05); however, no significant difference was found between the control and experimental groups (*p* > 0.05).

## 4. Discussion

The scientific community continues to investigate and debate the clinical efficacy of PRF in comparison to other biomaterials or conventional methodologies, despite its promising attributes. In particular, the biological mechanisms and effects of PRF on mineralized tissues have been the subject of numerous in vitro studies, particularly those that investigate its direct positive influence on mineralizing cells, such as osteoblasts [32,33].

In this study, the Linear Mixed Model was employed to compare lesion volumes between the control and experimental groups over time, while accounting for various confounding factors. The results indicate that the control group exhibited a notably lower lesion volume compared to the experimental group over time.

The Linear Mixed Model is particularly well-suited for this data type, as it can handle the correlations between repeated measurements on the same subjects [34]. By including time as a variable in the model, the Linear Mixed Model can assess how lesion volumes change over time and whether these changes differ between the control and experimental groups. This approach provides a more comprehensive understanding of the treatment’s effects and the progression of lesion volumes, accounting for individual variability and the influence of confounding factors.

Confounding factors are variables that can influence both the independent variable (treatment) and the dependent variable (lesion volume), potentially leading to biased results. By adjusting for these factors, the analysis aims to isolate the effect of the treatment itself. In this study, the confounding factors included baseline lesion volume, age, gender, vitamin D (D3+D2), Total Cholesterol, and classification of periradicular lesions. Adjusting for these factors, the Linear Mixed Model provides a clearer picture of the treatment’s effect on lesion volume, independent of these other influences. This adjustment helps ensure that the observed differences in lesion volume between the control and experimental groups are more likely due to the treatment itself rather than other variables. Both the main results of the Linear Mixed Model and linear regression suggest that the treatment administered to the experimental group did not effectively reduce the lesion size, as anticipated, highlighting the need for further investigation into the treatment’s efficacy and potential modifications.

Additionally, the analysis identified several confounding factors that significantly influenced periapical lesion volume. As expected, the baseline lesion volume played a critical role, as larger initial lesions were associated with larger volumes post-treatment. This finding is consistent with the principle that the initial severity of a condition often predicts the extent of recovery or progression.

Gender was particularly notable, with males showing a more markable lesion volume reduction than females. The investigation into the relationship between gender and the size of periapical lesions has yielded mixed results in the literature. While some studies suggest that males may experience larger periapical lesions compared to females, others indicate that females may have larger lesions, or that there is no significant difference between genders.

Sex differences in morphology and physiological function are evident in skeletal tissue, and these variations can influence the process of bone healing [35]. For instance, men typically have larger and stronger bones than women, which may make them more resistant to injury and fractures. Additionally, women face a significant increase in the risk of osteoporosis after menopause while, in men, the risk of developing osteoporosis gradually increases with age [36]. For example, Foroozandeh et al. reported that periapical lesions were more prevalent in males, attributing this difference to various biopsychosocial factors [37]; however, they did not specifically measure lesion size in relation to gender, leaving a gap in our understanding of whether the size of lesions differs significantly between males and females. Aysal et al. observed that periapical lesions were present in a comparable proportion of male and female patients but, again, this study did not provide direct comparisons of lesion sizes between genders [38]. Conversely, other studies indicated that females may present with larger periapical lesions. For example, Lucisano et al. showed that estrogen deficiency can alter the oral microbiota, leading to an increased bacterial presence and larger periapical lesions in females [39], but they did not specify gender differences in lesion size. This suggests that while lesion size is critical for treatment outcomes, the influence of gender on size remains understudied. Continuing to explore the general gender differences in bone healing, Ortona et al. have reported that males generally tend to experience more favorable healing outcomes compared to females, particularly in adulthood. This difference can be attributed to factors such as variations in sex hormone levels, the composition of the inflammatory response, and differences in cellular and molecular mechanisms. Additionally, lifestyle factors and behaviors also contribute to the observed variations in bone healing between sexes [40].

The limitations of this study are primarily related to the substantial variability observed in lesion volume. This variability could stem from inherent individual differences in the measured construct, measurement error, or the influence of unmeasured variables. Such variability reduced the statistical power to detect smaller effects and widened the confidence intervals for the regression coefficients. The relatively large standard deviation of the lesion volume, despite the observed statistical significance (*p* = 0.043) between research groups, raises concerns about the practical significance of the findings.

To address these limitations, future studies should consider larger sample sizes and more refined, standardized measurement techniques to enhance precision, reliability, and statistical power.

## 5. Conclusions

The results suggest that A-PRF may not offer a significant advantage in terms of reducing defect size or improving radiographic resolution.

## Figures and Tables

**Table 1:** Criteria for 2D Radiographic Healing Assessment.

**Score**	**Complete healing after endodontic surgery:**
	(A) Re-formation of periodontal space of normal width and lamina dura to be followed around the apex.
**1**	(B) Slight increase in width of apical periodontal space, but less than twice the width of non-involved parts of the root.
	(C) Tiny defect in the lamina dura (maximum 1 mm~) adjacent to the root filling.
	(D) Complete bone repair; bone bordering the apical area does not have the same density as surrounding non-involved bone.
	(E) Complete bone repair; no apical periodontal space can be discerned.
**Score**	**Incomplete healing (scar tissue)**
	Rarefaction has decreased in size or remained stationary, and is characterized by one or more of the following findings:
	(A) Bone structure are recognized within the rarefaction.
**2**	(B) The periphery of the rarefaction is irregular and may be demarcated by a compact bone border.
	(C) The rarefaction is located asymmetrically around the apex.
	(D) The connection of the rarefaction with the periodontal space is angular.
**Score**	**Uncertain healing**
	The rarefaction has decreased in size, and with one or more of the following characteristics:
	(A) The radiolucency is larger than twice the width of the periodontal space.
**3**	(B) The radiolucency is bordered by lamina-dura like bone structures.
	(C) The radiolucency has a circular or semicircular periphery.
	(D) The radiolucency is located symmetrically around the apex as a funnel-shaped extension of the periodontal spate.
	(E) Bony structures are discernible within the bony cavity.
**Score**	**Unsatisfactory healing**
**4**	The rarefaction has enlarged of is unchanged

**Table 2:** Criteria for 3D Radiographic Healing Assessment.

**Score**	**Complete healing**
	(A) Re-formation of periodontal space of normal width and lamina dura over the entire resected and un-resected root surfaces.
**1**	(B) Slight increase in width of apical periodontal space over the resected root surface, but less than twice the width of non-involved parts of the root.
	(C) Small defect in the lamina dura surrounding the root-end filling.
	(D) Complete bone repair with discernible lamina dura; bone bordering the apical area does not have the same density as surrounding non-involved bone.
	(E) Complete bone repair. Hard tissue covering the resected root-end surface completely. No apical periodontal space can be discerned.
**Score**	**Limited healing**
	Complete healing can be observed in immediate vicinity of the resected root surface, but the site demonstrates one of the following conditions:
	(A) The continuity of the cortical plate is interrupted by an area of lower density.
**2**	(B) A low density area remains asymmetrically located around the apex of has an angular connection with the periodontal space.
	(C) Bone has not fully formed in the area of the former access osteotomy.
	(D) In areas with pre-existing periodontal disease or physiologic fenestrations un-resected root surfaces do not demonstrate bone coverage and/or periodontal reattachment.
**Score**	**Uncertain healing**
	The volume of the low-density area appears decreased and demonstrates one of the following conditions:
**3**	(A) The thickness is larger than twice the width of the periodontal space.
	(B) The location is symmetrically around the apex as a funnel-shaped extension of the periodontal space.
**Score**	**Unsatisfactory healing**
**4**	The volume of the low-density area appears enlarged or unchanged

**Table 3:** Demographic and clinical characteristics of the study sample.

Variable	Control, *n* = 22	Experimental, *n* = 21	*p*-Value
Gender, *n* (%)			0.835
Male	8 (36.4)	7 (33.3)	
Female	14 (63.6)	14 (66.7)	
Age, mean (SD)	41.7 (13.7)	46.3 (14.4)	0.293
Tooth group, *n* (%)			0.519
C	1 (4.5)	2 (9.5)	
I	18 (81.8)	18 (85.7)	
P	3 (13.6)	1 (4.8)	
Jaw, *n* (%)			0.138
Md	1 (4.5)	4 (19.0)	
Mx	21 (95.5)	17 (81.0)	
Classification of periradicular lesions, *n* (%)			0.047
1	5 (22.7)	5 (23.8)	
2	3 (13.6)	1 (4.8)	
3	12 (54.5)	7 (33.3)	
4	0	7 (33.3)	
5	2 (9.1)	1 (4.8)	
D vitamin (D3+D2), mean (SD)	37.8 (15.3)	34.8 (15.2)	0.536
Total Cholesterol, mean (SD)	5.1 (1.48)	5.2 (0.87)	0.771
Volume before, mean (SD)	285.2 (306.3)	584.9 (764.7)	0.105
Volume after 6 months, mean (SD)	62.4 (90.8)	143.1 (182.7)	0.085
Volume after 12 months, mean (SD)	34.7 (63.4)	66.7 (80.5)	0.211

**Table 4:** Summary of Linear Mixed Model results, showing treatment and confounder effects on lesion volume (χ²(11) = 104.7, *p* < 0.001).

Parameters	Estimate	95% CI	*p*
Intercept	59.19	15.3–103.1	0.179
Groups			
Control-Experimental	−36.77	−72.35–-1.20	0.043
Baseline lesion volume, mm^3^	105.7	84.4–126.9	<0.001
Age, years	−17.6	−37.2–2.03	0.078
Vitamin D (D3+D2)	8.7	−8.8–26.3	0.322
Total Cholesterol	−5.5	−22.6–11.5	0.519
Gender			
Male–Female	−61.2	−101.4–-21.1	0.003
Classification of periradicular lesions			
C–B	−8.5	−57.0–40.0	0.726
D–B	61.2	1.9–120.6	0.043
E–B	−52.0	−117.3–13.3	0.116
F–B	−78.9	−141.8–-16.2	0.015

Note: CI, confidence interval; B, the lesion is limited to the periapical area; C, the lesion has eroded the buccal cortex; D, the lesion has eroded the lingual/palatal cortex; E, the lesion has eroded the lingual/palatal and buccal cortex, resulting in a through-and-through (tunnel) defect; F, an apico-marginal lesion is present, with complete denudation of the buccal root surface.

**Table 5:** The mean lesion volume after controlling for confounding effects.

	Control Group		Experimental Group			
	Mean (95% CI)	Marginal Mean * (95% CI)	Mean (95% CI)	Marginal Mean * (95% CI)	*p* ^1^	*p* ^2^
Baseline	285.2 (145.8–424.6)		584.9 (227.0–942.7)		0.228	
6 months	62.4 (19.9–104.9)	73.9 (29.5–118.0)	143.1 (57.6–228.6)	125.6 (80.6–171.0)	0.061	0.976
12 months	34.7 (3.1–66.2)	24.9 (0–52.3)	66.7 (22.1–111.2)	55.3 (25.3–85.2)	0.357	0.628

Note: CI, confidence interval; *, mean after controlling for age, gender, baseline lesion volume, vitamin D (D3+D2), Total Cholesterol, and classification of periradicular lesions; ^1^, result of mean comparison between groups; ^2^, result of mean comparison between groups after controlling for age, gender, baseline lesion volume, vitamin D (D3+D2), Total Cholesterol, and classification of periradicular lesions.

**Table 6:** Lesion assessment in control and experimental groups using both Molven’s and the modified Penn criteria methods.

		Experimental, *n* (%)		Control, *n* (%)			
		6 Months	12 Months	6 Months	12 Months	*p* ^1^	*p* ^2^
Molven’s Criteria	Complete healing after endodontic surgery	12 (60)	11 (73.3)	11 (55)	14 (77.8)	0.421	0.825
	Incomplete healing (scar tissue)	5 (25)	3 (20)	8 (40)	4 (22.2)		
	Uncertain healing	3 (15)	0	1 (5)	0		
	Unsatisfactory healing	0	1 (6.7)	0	0		
Penn Criteria	Complete healing	5 (25)	9 (60)	6 (30)	12 (66.7)	0.574	0.929
	Limited healing	10 (50)	3 (20)	12 (60)	4 (22.2)		
	Uncertain healing	5 (25)	2 (13.3)	2 (10)	2 (11.1)		
	Unsatisfactory healing	0	1 (6.7)	0	0		
	*p*-value	0.021	0.046	0.014	0.102		

Note: ^1^, comparison of data distribution between the experimental and control group at 6 months; ^2^, that at 12 months.

## Data Availability

Data presented in this study are available on request from the corresponding author.

## References

[1] Von Arx T. (2005). Failed root canals: The case for apicoectomy (periradicular surgery). J. Oral Maxillofac. Surg..

[2] von Arx T. (2011). Apical surgery: A review of current techniques and outcome. Saudi Dent. J..

[3] Setzer F.C., Shah S.B., Kohli M.R., Karabucak B., Kim S. (2010). Outcome of endodontic surgery: A meta-analysis of the literature—Part 1: Comparison of traditional root-end surgery and endodontic microsurgery. J. Endod..

[4] Anitua E., Sánchez M., Orive G., Andía I. (2007). The potential impact of the preparation rich in growth factors (PRGF) in different medical fields. Biomaterials.

[5] Liu T.J., Zhou J.N., Guo L.H. (2020). Impact of different regenerative techniques and materials on the healing outcome of endodontic surgery: A systematic review and meta-analysis. Int. Endod. J..

[6] Goyal B., Tewari S., Duhan J., Sehgal P.K. (2011). Comparative evaluation of platelet-rich plasma and guided tissue regeneration membrane in the healing of apicomarginal defects: A clinical study. J. Endod..

[7] Dhiman M., Kumar S., Duhan J., Sangwan P., Tewari S. (2015). Effect of Platelet-rich Fibrin on Healing of Apicomarginal Defects: A Randomized Controlled Trial. J. Endod..

[8] Dhamija R., Tewari S., Sangwan P., Duhan J., Mittal S. (2020). Impact of Platelet-rich Plasma in the Healing of Through-and-through Periapical Lesions Using 2-dimensional and 3-dimensional Evaluation: A Randomized Controlled Trial. J. Endod..

[9] Muresan G.C., Hedesiu M., Lucaciu O., Boca S., Petrescu N. (2022). Effect of Vitamin D on Bone Regeneration: A Review. Medicina.

[10] Diachkova E., Trifonova D., Morozova E., Runova G., Ashurko I., Ibadulaeva M., Fadeev V., Tarasenko S. (2021). Vitamin D and Its Role in Oral Diseases Development. Scoping Review. Dent. J..

[11] Zhang J., Hu W., Zou Z., Li Y., Kang F., Li J., Dong S. (2024). The role of lipid metabolism in osteoporosis: Clinical implication and cellular mechanism. Genes Dis..

[12] Krieger M. (1998). The “best” of cholesterols, the “worst” of cholesterols: A tale of two receptors. Proc. Natl. Acad. Sci. USA.

[13] Luegmayr E., Glantschnig H., Wesolowski G.A., Gentile M.A., Fisher J.E., Rodan G.A., Reszka A.A. (2004). Osteoclast formation, survival and morphology are highly dependent on exogenous cholesterol/lipoproteins. Cell Death Differ..

[14] Choukroun J., Khoury G., Khoury F., Russe P., Testori T., Komiyama Y., Sammartino G., Palacci P., Tunali M., Choukroun E. (2014). Two neglected biologic risk factors in bone grafting and implantology: High low-density lipoprotein cholesterol and low serum vitamin D. J. Oral Implant..

[15] Olivares-Navarrete R., Raines A.L., Hyzy S.L., Park J.H., Hutton D.L., Cochran D.L., Boyan B.D., Schwartz Z. (2012). Osteoblast maturation and new bone formation in response to titanium implant surface features are reduced with age. J. Bone Miner. Res..

[16] Fedele S., Sabbah W., Donos N., Porter S., D’Aiuto F. (2011). Common oral mucosal diseases, systemic inflammation, and cardiovascular diseases in a large cross-sectional US survey. Am. Hear. J..

[17] Gaetti-Jardim E.C., Santiago-Junior J.F., Goiato M.C., Pellizer E.P., Magro-Filho O., Jardim E.G. (2011). Dental implants in patients with osteoporosis: A clinical reality?. J. Craniofac. Surg..

[18] Sinibaldi R., Conti A., Sinjari B., Spadone S., Pecci R., Palombo M., Komlev V., Ortore M., Tromba G., Capuani S. (2017). Multimodal-3D imaging based on μMRI and μCT techniques bridges the gap with histology in visualization of the bone regeneration process. J. Tissue Eng. Regen. Med..

[19] Christiansen R., Kirkevang L.-L., Gotfredsen E., Wenzel A. (2009). Periapical radiography and cone beam computed tomography for assessment of the periapical bone defect 1 week and 12 months after root-end resection. Dentomaxillofac. Radiol..

[20] von Arx T., Janner S.F.M., Hänni S., Bornstein M.M. (2015). Agreement between 2D and 3D radiographic outcome assessment one year after periapical surgery. Int. Endod. J..

[21] von Arx T., Janner S.F., Hänni S., Bornstein M.M. (2016). Evaluation of New Cone-beam Computed Tomographic Criteria for Radiographic Healing Evaluation after Apical Surgery: Assessment of Repeatability and Reproducibility. J. Endod..

[22] Schloss T., Sonntag D., Kohli M.R., Setzer F.C. (2017). A Comparison of 2- and 3-dimensional Healing Assessment after Endodontic Surgery Using Cone-beam Computed Tomographic Volumes or Periapical Radiographs. J. Endod..

[23] Dawood A., Patel S., Brown J. (2009). Cone beam CT in dental practice. Br. Dent. J..

[24] Manacorda M., de Chaurand B.P., Merlone A., Tetè G., Mottola F., Vinci R. (2020). Virtual implant rehabilitation of the severely atrophic maxilla: A Radiographic Study. Dent. J..

[25] Yadav R., Mittal S., Tewari S., Gupta A., Duhan J., Sangwan P., Kumar V. (2022). Evaluation of amniotic membrane in the healing of apicomarginal defects using 2D and 3D imaging modalities: A randomized controlled trial. Quintessence Int..

[26] Grandi C., Pacifici L. (2009). The ratio in choosing access flap for surgical endodontics: A review. ORAL Implantol..

[27] Segura-Egea J.J., Gould K., Şen B.H., Jonasson P., Cotti E., Mazzoni A., Sunay H., Tjäderhane L., Dummer P.M.H. (2016). Antibiotics in Endodontics: A review. Int. Endod. J..

[28] Kim S., Kratchman S. (2006). Modern Endodontic Surgery Concepts and Practice: A Review. J. Endod..

[29] Dohan D.M., Choukroun J., Diss A., Dohan S.L., Dohan A.J., Mouhyi J., Gogly B. (2006). Platelet-rich fibrin (PRF): A second-generation platelet concentrate. Part I: Technological concepts and evolution. Oral Surg. Oral Med. Oral Pathol. Oral Radiol. Endodontol..

[30] Molven O., Halse A., Grung B. (1987). Observer strategy and the radiographic classification of healing after endodontic surgery. Int. J. Oral Maxillofac. Surg..

[31] Safi C., Kohli M.R., Kratchman S.I., Setzer F.C., Karabucak B. (2019). Outcome of Endodontic Microsurgery Using Mineral Trioxide Aggregate or Root Repair Material as Root-end Filling Material: A Randomized Controlled Trial with Cone-beam Computed Tomographic Evaluation. J. Endod..

[32] Goswami P., Chaudhary V., Arya A., Verma R., Vijayakumar G., Bhavani M. (2024). Platelet-Rich Fibrin (PRF) and its Application in Dentistry: A Literature Review. J. Pharm. Bioallied Sci..

[33] Johns D.A., Vidyanath S., Sam G., Shivashankar V.Y. (2013). Combination of platelet rich fibrin, hydroxyapatite and PRF membrane in the management of large inflammatory periapical lesion. J. Conserv. Dent..

[34] Jiming Jiang T.N. (2021). Linear and Generalized Linear Mixed Models and Their Applications.

[35] Seeman E. (2001). CLINICAL REVIEW 137 Sexual Dimorphism in Skeletal Size, Density, and Strength. J. Clin. Endocrinol. Metab..

[36] Black D.M., Cummings S.R., Melton L.J. (1992). Appendicular Bone Mineral and a Woman’s Lifetime Risk of Hip Fracture. J. Bone Miner. Res..

[37] Foroozandeh M., Ehsani A., Khazaei S. (2023). Assessment of Periapical Status in Posterior Root Canal-Treated Teeth Using Cone Beam Computed Tomography in the Iranian Population. Avicenna J. Dent. Res..

[38] Aysal Z., Kocasarac H.D., Orhan K., Helvacioglu-Yigit D. (2022). Radiological Assessment of Prevalance and Quality of Periapical Status of Endodontic Treatments. Med. Sci. Monit..

[39] Lucisano M.P., da Silva R.A.B., Pereira A.P.d.S., Romualdo P.C., Feres M., de Queiroz A.M., Nelson-Filho P., da Silva L.A.B. (2021). Alteration of the oral microbiota may be a responsible factor, along with estrogen deficiency, by the development of larger periapical lesions. Clin. Oral Investig..

[40] Ortona E., Pagano M.T., Capossela L., Malorni W. (2023). The Role of Sex Differences in Bone Health and Healing. Biology.

